# Additional right-sided upper “Half-Mini-Thoracotomy” for aortocoronary bypass grafting during minimally invasive multivessel revascularization

**DOI:** 10.1186/s13019-015-0334-6

**Published:** 2015-10-21

**Authors:** Hug Aubin, Payam Akhyari, Artur Lichtenberg, Alexander Albert

**Affiliations:** Department of Cardiovascular Surgery, Heinrich-Heine-University, Moorenstr. 5, Düsseldorf, 40225 Germany

**Keywords:** Coronary artery bypass grafting (CABG), Off-pump coronary artery bypass grafting (OPCAB), Minimally invasive coronary artery bypass grafting (MICS-CABG), Minimally invasive surgery, New surgical technique

## Abstract

**Background:**

Although minimally invasive coronary artery bypass grafting (MICS-CABG) has been shown to result in excellent clinical outcomes overall adoption rates still remain low. Traditional strategies for minimally invasive multivessel revascularization - usually performed through single-thoracotomy – have to deal with restricted grafting possibilities and possible increased susceptibility of arterial grafts to competitive flow, restraining their applicability to very specific indications or hybrid approaches and on top, are prone to conversion to full-sternotomy in case of left internal thoracic artery (LITA) insufficiency.

**Methods:**

Here, we present a novel alternative to the traditional MICS-CABG approaches by adding a right-sided upper “half-mini-thoracotomy”, which allows for aortocoronary bypass grafting in standard “off-pump” manner and adoption of similar revascularization principles as with conventional CABG during minimally invasive multivessel revascularization, though reducing restrictions inherent to current MICS-CABG strategies.

**Results:**

So far, feasibility and safety of this new approach has been successfully shown in 7 consecutive patients requiring surgical revascularization with no procedure-specific complications and graft configuration as well as intraoperative flow assessment comparable to those of similar patients operated via standard full-sternotomy off-pump coronary artery bypass (OPCAB) surgery.

**Conclusions:**

Further evaluation warranted, this technique might have the potential to develop into an additional approach for minimally invasive multivessel revascularization, especially in cases where competitive flow to arterial grafts is feared, while also serving as a bailout-strategy for traditional approaches in case of LITA insufficiency.

## Background

Despite the increasing numbers of percutaneous interventions, coronary artery bypass grafting (CABG) still remains the gold standard for the treatment of multivessel coronary artery disease in many patients. However, although clinical outcomes have dramatically improved since first introduction of CABG five decades ago, invasiveness of the procedure has not significantly changed, with most of the routinely performed CABG procedures still involving full-sternotomy and aortic clamping [1]. Although minimally invasive coronary artery bypass grafting (MICS-CABG) alternatives have been widely explored showing excellent clinical outcomes overall adoption rates still remain low.

Traditional strategies for minimally invasive multivessel revascularization are of high technical complexity - usually performed through a single-thoracotomy - and have to deal with restricted grafting possibilities and subsequent possible increased susceptibility of arterial grafts to competitive flow, restraining their applicability to very specific indications or hybrid approaches. On top, they are prone to conversion to full-sternotomy in case of left internal thoracic artery (LITA) insufficiency, due to lacking possibilities of aortocoronary venous bypass grafting.

Here, we present a sternotomy-free and clampless alternative to the standard CABG procedure, which allows for aortocoronary bypass grafting in standard “off-pump” manner, while reducing restrictions inherent to current minimally invasive cardiac surgery (MICS) CABG approaches.

## Methods

### Surgical technique description

**Fig. 1:**
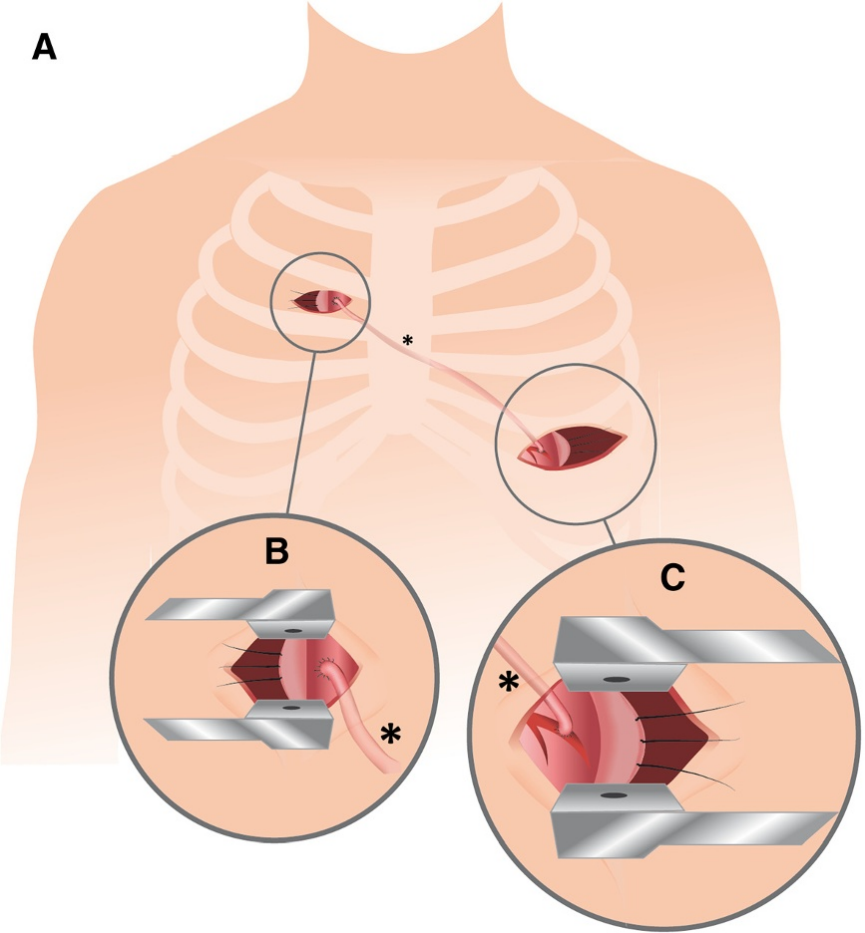
Schematics showing additional right-sided upper mini-thoracotomy for aortocoronary bypass grafting during minimally invasive multivessel revascularization. **a** Overview showing aortocoronary bypass course under intact sternum. **b** Proximal vein-graft anastomosis onto ascending aorta through a right-sided, anterior, parasternal mini-thoracotomy over the third intercostal space. **c** Distal vein-graft anastomosis onto target coronary vessel through a left-sided anterolateral mini-thoracotomy over the 5th-6th intercostal space. *aortocoronary bypass

The patient is prepped for surgery as if a minimally invasive direct coronary artery bypass (MIDCAB) procedure was to be performed [2] with addition of a double-lumen endotracheal tube for selective lung ventilation. Pleural cavity is accessed through a left-sided anterolateral mini-thoracotomy via an 8 cm incision over the 5th-6th intercostal space. The incision is performed more laterally than the traditional MIDCAB access in order to gain better exposition to the lateral and inferior portions of the heart. After left lung-deflation the pleural space is entered and the pericardium is obliquely dissected anterior to the phrenic nerve from the right outflow tract down to the apex and then anteriorly to the right cardiophrenic angle. Pericardial sutures are placed to facilitate exposure of targeted myocardial areas, while at the same time allowing for continuous ventilation of the left lung by retaining the left lower lobe. If target vessel exposition and ability to graft is satisfactory, the left internal thoracic artery (LITA) and the saphenous vein (SV) are harvested simultaneously. LITA anastomosis to primary target vessel is sewn in standard manner using an epicardial tissue stabilizer (Octopus™, *Medtronic*). Patient is tilted left-wards and access is gained to the ascending aorta through an additional, right-sided upper “half-mini-thoracotomy” via a parasternal 4 cm incision over the third intercostal space. After right lung-deflation the pleural space is entered and the pericardium is dissected transversally from the ascending aorta down to the right atrial appendage. Pericardial sutures are placed to facilitate exposure of the aortic landing zone, while at the same time allowing for continuous ventilation of the right lung by retaining the right upper lobe. One or multiple proximal vein-graft anastomoses can be hand-sewn in clampless manner using the *HeartString*™ device (*Guidant*). The blood-filled vein-graft(s) are tunneled under the sternum inside the pericardium to the target secondary vessel(s) (Fig. 1). Patient is tilted back to the initial position and distal anastomoses are sewn in standard manner under epicardial stabilization, verifying graft patency via transit time flow measurement (*VeriQ*™, *Medistim*). In case of hemodynamic instability or poor exposition the same procedure can be carried out under cardiopulmonary bypass (CPB) via standard femoral cannulation.

### Patients

So far, feasibility and safety of this approach has been successfully shown in 7 consecutive patients requiring surgical revascularization. 6 of the patients were elderly patients (mean age 76.3 ± 4.2 years) with no indication for extended arterial revascularization. The remaining patient was a 46-year old male, who firmly declined a conventional approach via sternotomy and in which we refrained from total arterial revascularization via standard multivessel small thoracotomy (MVST) approach because of expected increased susceptibility of the radial artery graft to competitive flow.

## Results

For bypass grafting the LITA (*n* = 7; 100 %) and an endoscopically harvested SV (*n* = 7; 100 %) were used, with a mean of 2.14 ± 0.35 distal anastomoses per patient (LAD, *n* = 7 (100 %); RCX marginal branch, *n* = 7 (100 %); RCA branch, *n* = 1 (14.3 %)). Proximal aortic anastomoses were handsewn via HeartString™ device in all 7 patients (100 %), while in one of the patients additional intraoperative proximal anastomoses revision via partial aortic clamping was necessary due to excessive graft length (14.3 %). In 5 of the patients (71.4 %) CPB via femoral cannulation was used for aortocoronary bypass grafting, mainly due to safety issues concerning the learning curve of implementing a new technique. Conversion rate to sternotomy was 0 % for all cases. All 7 patients (100 %) underwent complete revascularization (defined as revascularization of each major myocardial territory subtended by a coronary artery of 1.5 mm or more in diameter with stenosis ≥ 70 %) with no procedure-specific complications. In all patients, macroscopic anastomosis quality, graft configuration and intraoperative flow assessment were comparable to those of similar patients operated via standard full-sternotomy off-pump coronary artery bypass (OPCAB) surgery. Further, patient satisfaction at discharge in terms of having being able to avoid full-sternotomy, was particular high.

## Discussion

Evolving surgical strategies, such as OPCAB and MICS, are specifically developed to improve short- and long-term outcomes and to reduce the level of invasiveness of CABG. However, although prone to be beneficial for the patients in terms of reduced rates of transfusion and wound-infection as well as enhanced recovery to full activity and greater patient acceptance [3], overall adoption rates of MICS-CABG approaches remain low [1]. Usually performed through single-thoracotomy or port-access based they are either restrained to specific indications or hybrid approaches because of restricted grafting possibilities (MIDCAB), have to deal with increased susceptibility of arterial grafts to competitive flow [4] (MVST), need specialized infrastructure and training (totally endoscopic coronary artery bypass - TECAB) and are usually prone to conversion to full-sternotomy in case of LITA insufficiency.

Although, minimally invasive multivessel revascularization with aortocoronary bypass grafting performed through single-thoracotomy [5] is possible and has been shown to result in excellent clinical outcomes [6] as well as angiographic graft patency [7] adoption rates remain low. Here, the technical complexity - demanding intricate exposure maneuvers to anastomose grafts onto the ascending aorta while being highly dependent on favorable patient anatomy – is probably one of the major concerns in surgeons not experienced with this approach, restraining the applicability of MICS-CABG surgery to a small number of surgeons and patients.

As demonstrated in this report, an additional right-sided upper “half-mini-thoracotomy” can be an easy to adopt alternative to current MICS-CABG approaches, allowing for aortocoronary bypass grafting in standard OPCAB manner and liberal adoption of similar revascularization principles as with conventional CABG. Although this means having an additional “small scar” on the upper right thorax, patients can hereby potentially be spared a full-sternotomy without need to compromise in the number of employed grafts.

## Conclusion

Further evaluation warranted, this technique might have the potential to develop into an additional approach for minimally invasive multivessel revascularization, especially in cases where competitive flow to arterial grafts is feared, while also serving as a bailout-strategy for traditional approaches in case of LITA insufficiency.

## Abbreviations

CABG: Coronary artery bypass grafting
CPB: Cardiopulmonary bypass
LAD: Left anterior descending coronary artery
LITA: Left internal thoracic artery
MICS: Minimally invasive cardiac surgery
MICS-CABG: Minimally invasive coronary artery bypass grafting
MIDCAB: Minimally invasive direct coronary artery bypass
MVST: Multivessel small thoracotomy
OPCAB: Off-pump coronary artery bypass
RCA: Right coronary artery
RCX: Circumflex coronary artery LAD
SV: Saphenous vein
TECAB: Totally endoscopic coronary artery bypass
